# IgG-based B7-H3xCD3 bispecific antibody for treatment of pancreatic, hepatic and gastric cancer

**DOI:** 10.3389/fimmu.2023.1163136

**Published:** 2023-04-14

**Authors:** Martina S. Lutz, Latifa Zekri, Laura Weßling, Susanne Berchtold, Jonas S. Heitmann, Ulrich M. Lauer, Gundram Jung, Helmut R. Salih

**Affiliations:** ^1^Department of Internal Medicine, Clinical Collaboration Unit Translational Immunology, German Cancer Consortium (DKTK), University Hospital Tuebingen, Tuebingen, Germany; ^2^Cluster of Excellence iFIT (EXC 2180) “Image-Guided and Functionally Instructed Tumor Therapies”, University of Tuebingen, Tuebingen, Germany; ^3^Department of Immunology, Eberhard Karls Universität Tübingen, Tuebingen, Germany; ^4^Department of Internal Medicine VIII, Medical Oncology & Pneumology, University Hospital Tübingen, Tuebingen, Germany; ^5^German Cancer Research Center (DKFZ), German Cancer Consortium (DKTK), Tübingen, Germany

**Keywords:** immunotherapy, pancreatic cancer, hepatic cancer, gastric cancer, B7-H3 (CD276), CD3, bispecific antibody, gastrointestinal cancer

## Abstract

T cell-based immunotherapy has significantly improved treatment options for many malignancies. However, despite these and other therapeutic improvements over the last decades, gastrointestinal cancers, in particular pancreatic, hepatic and gastric cancer, are still characterized by high relapse rates and dismal prognosis, with an accordingly high unmet medical need for novel treatment strategies. We here report on the preclinical characterization of a novel bispecific antibody in an IgG-based format termed CC-3 with B7-H3xCD3 specificity. In many cancer entities including pancreatic, hepatic and gastric cancers, B7-H3 (CD276) is overexpressed on tumor cells and also on the tumor vasculature, the latter allowing for improved access of immune effector cells into the tumor site upon therapeutic targeting. We demonstrate that CC-3 induces profound T cell reactivity against various pancreatic, hepatic and gastric cancer cell lines as revealed by analysis of activation, degranulation and secretion of IL2, IFNγ as well as perforin, resulting in potent target cell lysis. Moreover, CC-3 induced efficient T cell proliferation and formation of T cell memory subsets. Together, our results emphasize the potential of CC-3, which is presently being GMP-produced to enable clinical evaluation for treatment of pancreatic, hepatic and gastric cancer.

## Introduction

Malignancies of the gastrointestinal tract belong to the most prevalent cancers, accounting for 26% of global cancer incidence and 35% of all cancer-related deaths (1). Current treatment strategies include chemotherapy, surgery, radiotherapy and targeted therapies. Despite therapeutic improvements over the last decades, among others due to incorporation of antibody-based approaches like monoclonal antibodies (mAbs) targeting e.g. HER2 or VEGF (2, 3), the still high relapse rates and dismal prognosis underline the high medical need for new therapeutic strategies.

In the last decade, immunotherapeutic approaches to mobilize T cells against tumor cells have significantly improved oncological treatment options. In particular, immune checkpoint inhibition (ICI) has become a mainstay of treatment in many solid tumor entities (4, 5). However, long-term remissions are still rare and many patients do not respond to treatment. Chimeric antigen receptor (CAR) T cell therapy and bispecific antibodies (bsAbs) have shown remarkable success in the treatment of hematological malignancies (6, 7) but are so far not effective in solid tumors.

B7-H3 (CD276) is a type I transmembrane protein belonging to the B7 family of immune checkpoint proteins (8). Due to rather specific expression on a wide array of cancer entities, B7-H3 presently receives high interest as target for immunotherapeutic approaches (9, 10). Notably, B7-H3 is not only expressed on tumor cells but also on the tumor vasculature (11). The latter may facilitate improved access of immune effector cells to solid tumors upon therapeutic targeting, thereby overcoming the lacking access of immune effector cells to the tumor site, a major obstacle for T cell-based immunotherapy of solid tumors. Based on previous work including development of bsAbs with FLT3xCD3 (12) and PSMAxCD3 (13, 14) specificity until the stage of clinical evaluation (NCT05143996, NCT04104607 and NCT04496674), we here preclinically evaluated a B7-H3xCD3 bsAb termed CC-3, which is presently undergoing GMP production, for treatment of pancreatic, hepatic und gastric cancer.

## Materials and methods

### Production and purification of bispecific antibodies

B7-H3xCD3 and its isotype control MOPCxCD3 were generated as described previously (13). In brief, the constructs were produced in ExpiCHO cells (Gibco, Carlsbad, CA, USA) and purified from culture supernatant by affinity chromatography on Mabselect affinity columns (GE Healthcare, Munich, Germany) followed by analytical and preparative size exclusion chromatography using Superdex S200 Increase 10/300GL and HiLoad 16/60 columns (GE Healthcare). Endotoxin levels were measured with EndoZyme II (BioMerieux, Marcy-l’Étoile, France) according to the manufacturer’s instructions and < 0.5 EU/ml.

### Cells

All cell lines were from ATCC (American Type Culture Collection) and were selected to best reflect the respective cancer entities. Hep3B and SNU387 represent hepatocellular carcinoma, the most common form of liver cancer. The pancreatic cancer cell lines were derived from adenocarcinomas, the most common type of pancreatic cancer. Of these, PANC-1 cells represent pancreatic ductal carcinoma, the most common subtype of pancreatic adenocarcinoma. Adenocarcnimoa also is the most common type of gastric cancer, with NCI-N87 and MKN-45 cells representing well differentiated Lauren intestinal-type gastric adenocarcinoma and poorly differentiated Lauren diffuse-type gastric adenocarcinoma, respectively. Cells were tested routinely for mycoplasma contamination every three months. Authenticity was determined on a regular basis by validating the respective immunophenotype described by the provider using flow cytometry. Peripheral Blood Mononuclear cells (PBMC) of healthy donors were isolated by density gradient centrifugation (Biocoll; Biochrom, Berlin, Germany), and monocytes within the PBMC were depleted for coculture experiments using human CD14 MicroBeads UltraPure kit (Miltenyi Biotec, Bergisch Gladbach, Germany). Where indicated, T cells within PBMC were isolated by using either Pan T cell Isolation Kit, human CD4 Micro Beads or human CD8 Micro Beads (Miltenyi Biotec).

### Relative gene expression of CD276 based on TCGA database analysis

Data on relative CD276 expression in tumor tissue of pancreatic, hepatic and gastric cancer patients was obtained from the Cancer Genome Atlas (TCGA) database and the GTEx project utilizing the Gene Expression Profiling Interactive Analysis (GEPIA) web server as described previously. The datasets PAAD LIHC and STAD were downloaded from TCGA (http://www.oncolnc.org/) and analyzed using the online web server GEPIA (http://gepia.cancer-pku.cn/).

### Immunofluorescence

Tumor cells were incubated with monocyte-depleted PBMC of healthy donors (E:T 4:1), treated for 3 h and subsequently fixed in 4% paraformaldehyde (PFA) in PBS for 10 min at -20°C. Cells were then blocked using 5% BSA, 0.2% Triton X-100, 0.1% Tween for 60 min, washed three times with PBST (PBS + 0.1% Tween20) and stained using a rabbit anti-human-α-Tubulin antibody (clone 11H10, Cell Signaling, Denvers, MA) and a murine anti-human-Perforin antibody (clone δG9, BD Pharmingen, Heidelberg, Germany), followed by an Alexa-Fluor 488 labelled anti-mouse and Alexa-Fluor 594 labelled anti-rabbit antibody (Invitrogen, Waltham, MA). Slides were mounted in fluorescent mounting medium; DAPI was used for counter-staining. Pictures were acquired using a Zeiss 800 inverse laser scanning microscope (Zeiss, Oberkochen, Germany) and images were processed using ImageJ (15).

### Flow cytometry

For analysis of B7-H3 surface expression and B7-H3xCD3 binding, cells were stained with a parental murine B7-H3 antibody (10 µg/mL) carrying the same B7-H3 binding clone as our construct (7C4), B-H3xCD3 or the corresponding isotype controls followed by a goat anti-mouse-PE conjugate (Dako, Glostrup, Denmark) or a donkey anti-human-PE conjugate (Jackson ImmunoResearch, West Grove, USA), respectively. T cell activation, degranulation and proliferation were determined using CD69-PE, CD107a-PE (BD Pharmingen) as well as CD4-APC, CD8-FITC, CD62L-PB and CD45ro-PeCy7 (BioLegend, San Diego, CA) fluorescence conjugates. For flow cytometric analysis of target cell lysis, tumor cells were loaded with 2.5 µM CellTrace™ Violet (Thermo Fisher Scientific, Waltham, MA) and cultured with monocyte-depleted PBMC (E:T 4:1) in the presence or absence of B7-H3xCD3 or MOPCxCD3 (1 nM each). Standard calibration beads (Sigma-Aldrich, St. Louis, MO) were used to ensure analysis of equal assay volumes and to account for the number of target cells that had vanished from the culture. 7AAD (Biolegend) was used for live- and dead-cell discrimination. Measurements were performed using a FACS Canto II or FACS Fortessa (BD Biosciences, San Diego, CA) and data was analyzed using the software FlowJo (FlowJo LCC, Ashland, OR).

### T cell activation and degranulation assays

To determine activation and degranulation in the presence of target cells, tumor cells were cultured with monocyte-depleted PBMC of healthy donors (E:T 4:1) in the presence or absence of B7-H3xCD3 or MOPCxCD3 (1 nM each). For analysis of T cell activation, CD69 expression was determined after 24 h. To compare activation of T cells within PBMC preparations to isolated T cell subpopulations in the absence of target cells, 1 µg/ml recombinant human B7-H3 was coated on 96-well plates overnight. Subsequently, PBMC preparations or isolated T cells and MOPCxCD3 or B7-H3xCD3 (5 nM each) were added, incubated for 72 h and analyzed by flow cytometry for CD69 expression. To analyze T cell degranulation, cells were cultured for 4 h in the presence of CD107a-PE (1:25), BD GolgiStop and BD GolgiPlug (1:1000, both BD Biosciences). Analysis was conducted using flow cytometry.

### T cell proliferation assays

For long-term proliferation assays, monocyte-depleted PBMC were loaded with 2.5 µM CellTrace™ Violet cell proliferation dye (Thermo Fisher Scientific) and incubated with tumor cells (E:T 4:1) and the indicated bsAbs (1 nM each). On day 3, cells were reincubated with fresh target cells and treatment was repeated. On day 6, proliferation of CD4^+^ and CD8^+^ T cells was analyzed by flow cytometry. For T cell subset analysis, PBMC were incubated with tumor cells (E:T 1:1) and the indicated bsAbs (1 nM each). On day 3, cells were reincubated with fresh target cells and treatment. On day 6, T cell subsets were determined by flow cytometric analysis for expression of CD4, CD8, CD45ro and CD62L.

### Analysis of cytokine secretion

Monocyte-depleted PBMC of healthy donors were cultured with tumor cells (E:T ratio 4:1) in the presence or absence of bsAb or control (1 nM each). After 24 h, supernatants were collected and analyzed for IL-2, IFNγ, IL-10 and TNF using Legendplex assays (BioLegend).

### Cytotoxicity assays

Monocyte-depleted PBMC of healthy donors were cultured with tumor cells (E:T ratio 4:1) in the presence or absence of bsAb or control (1 nM each). Real-time cytotoxicity analysis was conducted using the xCELLigence RTCA system (Roche Applied Science, Penzberg, Germany).

### Statistics

If not otherwise indicated, values depict means ± standard deviation (SD). Student’s t test, Mann‐Whitney U test, one‐way ANOVA and Friedman’s test was used for continuous variables. If significant differences by ANOVA were found, group wise comparison was done (Tukey’s multiple comparison test). If significant differences were found by Friedman’s test, Dunn’s multiple comparisons test was used. All statistical tests were considered statistically significant when *p* was below 0.05. Statistical analysis was performed using GraphPadPrism (v.8.1.0).

## Results

### Characterization of B7-H3 expression and binding of the B7-H3xCD3 bsAb CC-3 in pancreatic, hepatic and gastric cancer cell lines

**Figure 1:**
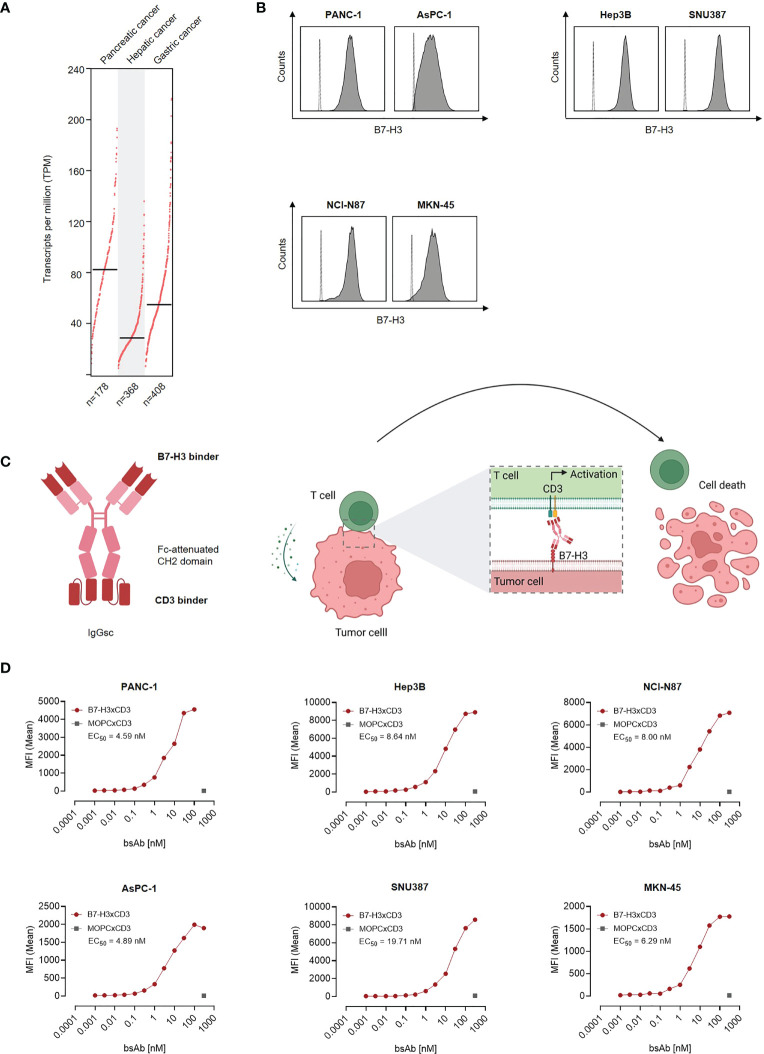
Characterization of B7-H3 expression and binding of CC-3 in gastrointestinal cancer cell lines. **(A)** CD276 gene expression profile in pancreatic, hepatic and gastric cancer was analyzed using the online web server GEPIA. **(B)** The indicated cancer cell lines were stained using a murine monoclonal B7-H3 antibody (clone 7C4) followed by an anti-mouse PE conjugate and analyzed using flow cytometry. B7-H3 expression on pancreatic cell lines AsPC-1 and PANC-1, hepatic cell lines Hep3B and SNU-387 and gastric cell lines MKN-45 and NCI-N87 is shown (shaded peaks, anti-B7H3; open peaks, control). **(C)** Schematic illustration and mechanism of action of the B7-H3xCD3 bsAb CC-3. The graphic was created by BioRender (BioRender.com, Toronto, Canada). **(D)** The indicated tumor cells were incubated with increasing concentrations of CC-3 or the respective isotype control MOPCxCD3, followed by an anti-human PE conjugate. Binding of the constructs to the indicated cell lines was analyzed by flow cytometry. MFI, mean fluorescence intensities.

As a first step, B7-H3 mRNA expression was studied by analysis of TCGA data sets of 178 pancreatic adenocarcinomas, 368 hepatocellular carcinomas and 408 gastric adenocarcinomas. Highest mRNA expression was observed in pancreatic adenocarcinoma, followed by gastric adenocarcinoma and hepatocellular carcinomas (**Figure 1A**). Next we characterized whether and to what extent B7-H3 was expressed on the surface of pancreatic, hepatic and gastric cancer cell lines. To this end we used the B7-H3 monoclonal antibody clone 7C4 that also served as target binder in our B7-H3xCD3 bsAb. Flow cytometric analysis revealed substantial B7-H3 expression in all tested cell lines (**Figure 1B**). Next we studied binding of our B7-H3xCD3 bsAb CC-3, which contains the variable domain of 7C4 cloned into our previously described IgGsc bsAb format (13), with the single chain sequence of UCHT-1 carrying four mutations in the CDR-H2 and one mutation in the FR-H3 resulting in reduced affinity to CD3 (clone M18) as effector part (**Figure 1C**) (16). Binding titration experiments using the indicated pancreatic, hepatic and gastric cell lines revealed EC_50_ values between 4.59 nM and 19.71 nM (**Figure 1D**), whereas no unspecific binding to B7-H3-negative cells was observed (**Figures S1A, B**).

### Induction of T cell activation

**Figure 2:**
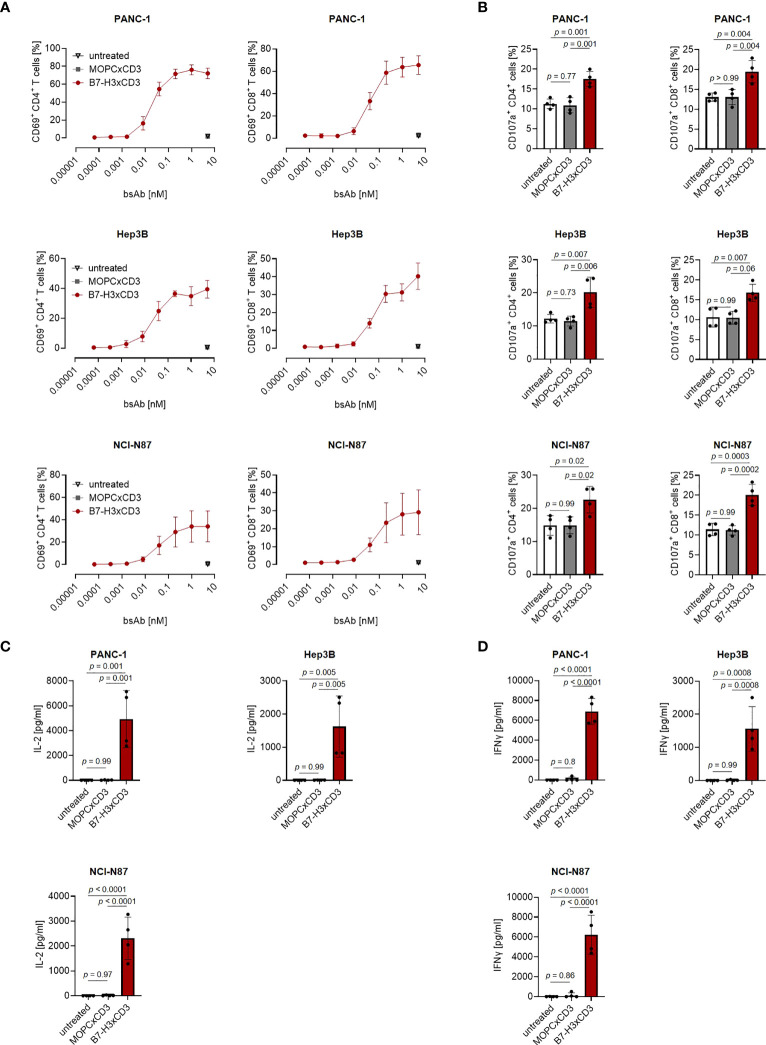
Induction of T cell activation against gastrointestinal cancer cell lines by CC-3. Monocyte-depleted PBMC of healthy donors were incubated with the indicated tumor cell lines (E:T 4:1) in the presence or absence of CC-3 or MOPCxCD3. If not stated otherwise, all constructs were used at 1 nM. **(A)** Activation of CD4+ and CD8+ T cells was determined by flow cytometric analysis for CD69 expression after 24 hours. Combined data obtained with PBMC of three independent donors are shown. **(B)** Degranulation of CD4+ and CD8+ T cells was determined by expression of CD107a after 4 h. Combined data obtained with PBMC of four independent donors are shown. **(C)** IL-2 and **(D)** IFNγ levels in culture supernatants were measured after 24 h using LEGENDplex assays. Combined data obtained with PBMC of four independent donors are shown. E:T, effector to target; PBMC, peripheral blood mononuclear cell.

**Figure 3:**
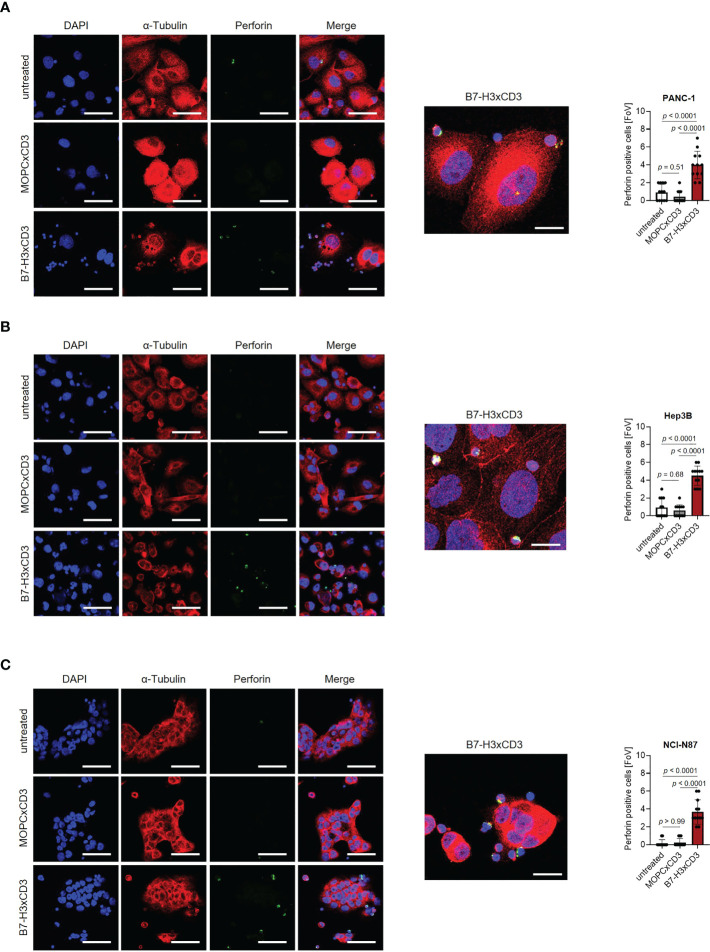
Induction of Perforin by CC-3. Monocyte-depleted PBMC of healthy donors were incubated with target cells (E:T 4:1) in the presence or absence of CC-3 or MOPCxCD3 (1 nM each) for 3 hours and subsequently stained for Perforin (green) and α-Tubulin (red). DAPI was used for counterstaining. Scale bars of left panels indicate 50 µM, original magnification x40. Scale bar of exemplary immunofluorescence staining indicates 20 µm, original magnification x63. Perforin positive cells were quantified per FoV (n=12) out of three independent experiments. Results obtained with **(A)** PANC-1, **(B)** Hep3B and **(C)** NCI-N87 cells are shown. DAPI, 4′,6-diamidino-2-phenylindole; E:T, effector to target; FoV, field of view.

Next, we determined the capacity of CC-3 to induce T cell reactivity against pancreatic, hepatic and gastric cancer cell lines. PBMC of healthy donors were depleted of monocytes that can express substantial levels of B7-H3 upon activation (8, 9) and cultured with the indicated target cells in the presence or absence of increasing concentrations of CC-3 or the isotype control MOPCxCD3. Flow cytometric analysis of CD69 expression revealed maximal activation of CD4^+^ and CD8^+^ T cells with CC-3 concentrations as low as 1 nM CC-3 (**Figure 2A**). Use of the above described PBMC preparations and analysis of the effects of CC-3 on T cells therein served to most closely reflect the physiological situation in patients, where other components of the blood may influence bsAb-induced T cell immunity (17, 18). Analyses evaluating the effect of immobilized CC-3 or isotype control in the absence of tumor targets revealed comparable activation of T cells within PBMC preparations and isolated T cells and excluded potential confounders by the tumor cells. (**Figure S2**). Analysis of T cell degranulation as determined by analysis of CD107a expression confirmed that CC-3 potently stimulated T cells with maximum effects observed already with doses of 1nM (**Figure 2B**). No effects were observed when the isotype control MOPCxCD3 or the B7-H3 negative HL-60 cells as targets were used, confirming strictly target antigen-restricted activity of our construct (**Figures 2A, B**; **S1C, D**). In line, analysis of culture supernatants by Legendplex assays showed a significant increase in IL-2, IFNγ, IL-10 and TNF secretion after treatment with 1 nM CC-3, but not with the isotype control or when target antigen negative tumor cells were used (**Figures 2C, D**; **S1E**, **S3**). When we used immunofluorescence staining to visualize induction of T cell reactivity against target cells, a significantly higher number of Perforin positive cells within the coculture was observed in samples treated with CC-3 compared to untreated samples. No effect was observed with the isotype control, again confirming target cell restricted activity of our bsAb CC-3 (**Figures 3A–C**; **S4**).

### Induction of T cell proliferation

**Figure 4:**
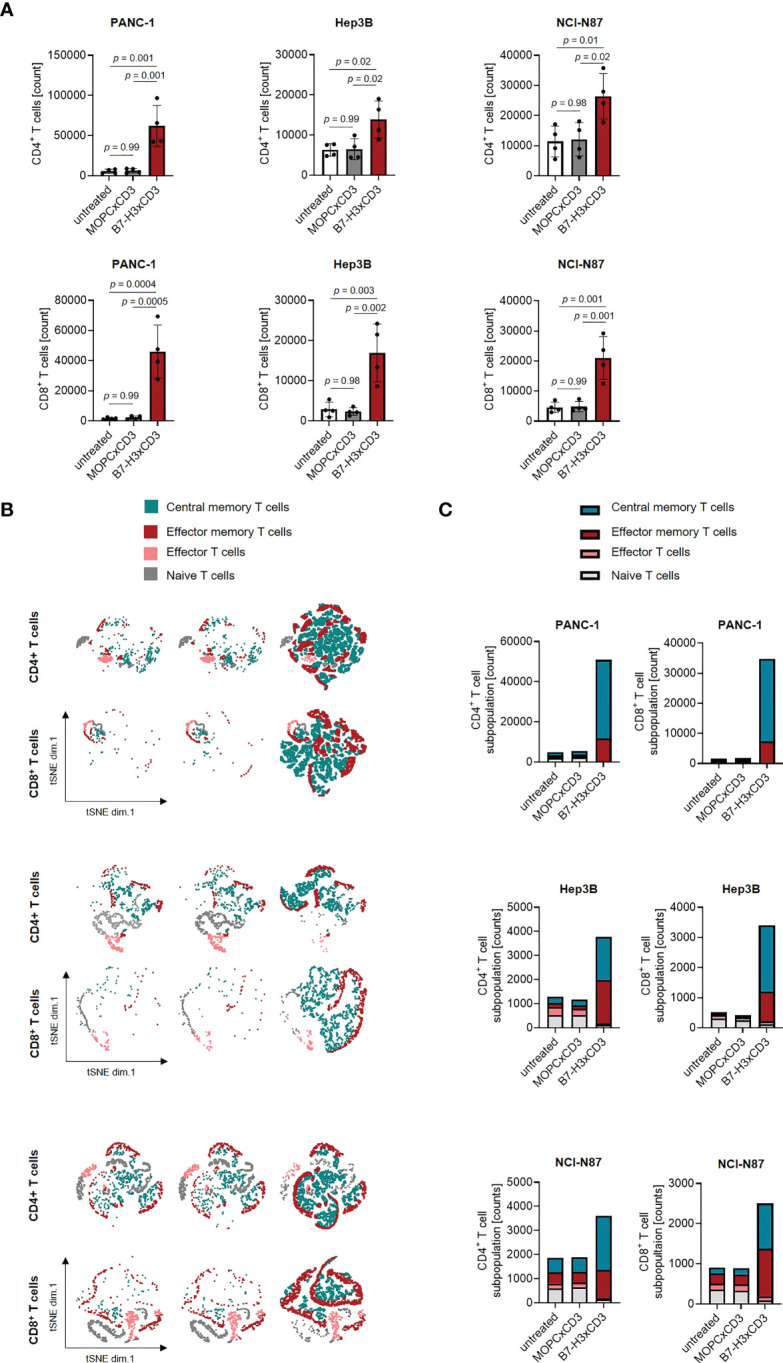
Induction of T cell proliferation and memory T cell populations by CC-3. **(A)** Monocyte-depleted PBMC of healthy donors (n=4) were labelled with CellTrace™ Violet cell dye and incubated with or without MOPCxCD3 or CC-3 (1 nM each) in the presence of PANC-1, Hep3B, or NCI-N87 cells (E:T 4:1). After 72 h, PBMC were reexposed to fresh target cells and the respective treatment for additional 72 h. On day 6, proliferation was determined by flow cytometry. **(B, C)** PBMC of healthy donors (n=5) were incubated with or without MOPCxCD3 or CC-3 (1 nM each) in the presence of PANC-1, Hep3B, or NCI-N87 cells (E:T 1:1). After 72 h, cells were reexposed to fresh target cells and the respective treatment. On day 6, subpopulations of CD4^+^ and CD8^+^ T cells were determined by flow cytometric analysis. Effector T cells were defined as CD62L^-^CD45ro^-^, naive T cells as CD62L^+^CD45ro^-^, effector memory T cells as CD62L^-^CD45ro^+^ and central memory T cells as CD62L^+^CD45ro^+^. **(B)** representative t-distributed stochastic neighbor embedding (tSNE) plots and **(C)** pooled data are shown. E:T, effector to target. PBMC, peripheral blood mononuclear cell.

As induction of T cell proliferation is an important prerequisite to combat high tumor burden, we next labelled PBMC using CellTrace™ Violet and analyzed induction of CD4^+^ and CD8^+^ T cell counts (**Figures 4A–C**) and cell dye dilution (**Figure S5**) in cocultures with gastric, hepatic and pancreatic tumor cells upon treatment with 1 nM of CC-3 using flow cytometry. We observed profound T cell proliferation and significantly increased T cell counts in samples treated with CC-3, but not upon application of isotype control or when target cells were negative for B7-H3 (**Figures 4A**; **S1F**). As memory T cells constitute the subset most relevant for therapeutic success (19, 20), we next analyzed which T cell subsets were proliferating and found that CC-3 preferentially induced expansion of effector memory and central memory T cells in a target cell restricted manner (**Figures 4B, C**; **Figure S1G**).

### Induction of target cell lysis

**Figure 5:**
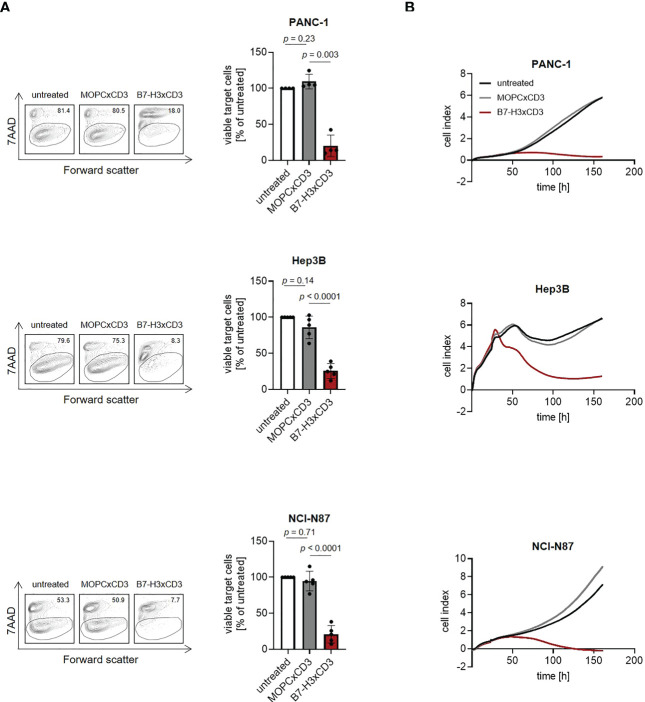
Induction of tumor cell lysis mediated by CC-3. Monocyte-depleted PBMC of healthy donors were incubated with the indicated target cells at an E:T ratio of 4:1 in the presence or absence of MOPCxCD3 or CC-3 (1 nM each). **(A)** Lysis of PANC-1, Hep3B and NCI-N87 cells was determined by flow cytometry-based lysis assay after 72 h. Left panels depict exemplary results right panels show combined data obtained with PBMC of five independent donors. **(B)** Long-term cytotoxic effects of PBMC of healthy donors (n=4) against PANC-1, NCI-N87 and Hep3B cells was determined using xCELLigence system. E:T, effector to target.

Finally, we analyzed whether induction of T cell activation and proliferation by CC-3 treatment were mirrored by a corresponding effect regarding lysis of pancreatic, hepatic and gastric tumor cells. PBMC were cocultured with target cells and treated with CC-3 or isotype control (1 nM). Flow cytometry-based lysis assays revealed significant induction of cytotoxicity against pancreatic, hepatic and gastric tumor cells by CC-3 after 72 h. Again, isotype control had no effect, and analyses with the B7-H3xCD3 negative HL-60 cells confirmed target antigen-specific efficacy (**Figures 5A**; **S1H**, **S6**). Notably, analyses using the xCELLigence system confirmed the ability of CC-3 to mediate target efficacy of CC-3 over extended time periods, which indicates that CC-3 may induce sustained anti-tumor efficacy (**Figure 5B**).

## Discussion

Although therapeutic options for solid tumors have overall improved over the last decades, treatment of gastrointestinal cancers remains still challenging. Several small molecule inhibitors have been approved by the US Food and Drug Administration (FDA) as therapeutic option for hepatic, gastric and pancreatic cancer, but the benefit of these therapies is limited (21–25). Antibody-based approaches targeting angiogenesis-(e.g., Ramucirumab) or oncogenic signaling pathways (e.g., EGF Cetuximab or HER2 Trastuzumab) likewise show only limited benefit. T cell based-immunotherapy, especially ICI, which has revolutionized oncological treatment e.g. of lung and skin cancer, has also been evaluated for treatment of metastasized GI cancer, but except for the small group with microsatellite instability, achieves only limited efficacy (26–31). Especially in pancreatic cancer, ICI is far from being sufficiently effective, in large part likely due to an immunosuppressive tumor microenvironment (32). Moreover, the dense extracellular matrix of pancreatic cancer may act as physical barrier that prevents tumor infiltration by T and B lymphocytes (33). So far, limited evidence is available that ICI may be effective in some patients with hepatocellular carcinoma, but a reliable predictor for treatment response, e.g. characteristics of the tumor environment, has not been identified yet (34). Overall, new therapeutic concepts to improve treatment options of GI cancer patients are urgently needed. In the present study, we report on the preclinical characterization of a bsAb targeting B7-H3 and CD3 for treatment of gastric, hepatic and pancreatic cancer.

Compared to ICI, bsAbs and CAR T cells induce T cell antitumor immunity in a more targeted manner. Other than CAR T cells, bsAbs constitute readily available ‘off the shelf´ reagents, avoiding the delay of treatment that is required for the production of CAR T cells and contributes to their vast costs upon clinical application. CAR T cells and bsAbs share the shortcoming that their therapeutic success so far is limited to hematologic malignancies. The reasons are not yet fully understood, but limited access of T cells to the tumor site appears to constitute a major obstacle (35, 36). In our view, targeting both tumor cells and tumor vessels thus seems critical, the latter allowing for sufficient influx of immune cells to the tumor site across a damaged or inflamed endothelial barrier. This reasoning is supported by reports demonstrating that even high numbers of tumor-specific T cells fail to induce sufficient antitumor responses unless a proinflammatory tumor environment has been generated (37, 38).

B7-H3 has attracted considerable interest as promising target for cancer immunotherapy because it is not only overexpressed on tumor cells in various types of cancer including GI cancer (9, 39, 40), but also on tumor-associated endothelial and stromal cells (41, 42). High expression of B7-H3 on tumor tissue is associated with poor clinical outcome and lymph node metastasis (43), and several studies report first evidence that B7-H3 positive tumor cell fractions may be enriched for cancer stem cells (44–46). In addition, B7-H3 is reported to act as coinhibitory receptor in the B7-CD28 pathway, suppressing T cell antitumor immunity (47–49). Specifically in pancreatic cancer, B7-H3 was reported to promote tumor progression, and its inhibition reduced cancer metastasis *in vivo* (39). In hepatocellular carcinoma, high B7-H3 expression was associated with adverse clinicopathologic features and poor outcome (50). Another study demonstrated that B7-H3 promotes gastric cancer cell migration and invasion (51). We have recently reported on the preclinical characterization of an optimized B7-H3xCD3 bsAb and showed that targeting a membrane-proximal B7-H3 epitope allows for reduction of CD3 affinity while maintaining therapeutic efficacy. In analyses with colorectal cancer cells, our lead compound CC-3 demonstrated superior tumor cell killing, T cell activation, proliferation, and memory formation, while undesired cytokine release was reduced (16). Based on these characteristics and the aforementioned data on the expression and pathophysiological involvement of B7-H3 we reasoned that CC-3 would also constitute a promising compound for other gastrointestinal cancer entities. Initial flow cytometric analyses revealed that indeed high B7-H3 levels were expressed in all tested pancreatic, hepatic and gastric cancer cell lines, confirming suitability as therapeutic target. Subsequent functional characterization documented strong induction of T cell activation, degranulation and secretion of the “antitumor cytokines” like IFNγ and IL-2 as well as potent induction of tumor cell lysis. In addition, we could demonstrate that CC-3 potently induces T cell proliferation, which is a critical prerequisite to enable treatment of patients with higher disease burden. In this context it is particularly noteworthy that CC-3 triggered mainly the proliferation of memory T cells, which are considered to be crucial for therapeutic success (19, 20). These findings support and extend our recently reported data for CC-3 in CRC (16). Of note, various factors like the tumor microenvironment and T cell exhaustion influence the therapeutic success of bsAb treatment and are not accounted for in our experimental systems. Additional preclinical investigations to address the influence of these and other confounders of treatment efficacy are warranted, and eventually could be correlated with findings upon clinical evaluation.

Several approaches targeting B7-H3 are currently under clinical investigation, reflecting the growing interest in this target for antibody-based immunotherapy. This includes among others CAR T cell-based therapies (NCT04897321, NCT05211557, NCT05341492, NCT04483778, NCT05323201, NCT05241392, NCT05474378, NCT03198052, NCT04185038, NCT05366179, NCT04670068, NCT05143151) which are being evaluated in various tumor entities including hepatocellular carcinoma and advanced pancreatic carcinoma.

In conclusion, the preclinical data reported in this study documenting efficacy of CC-3 in pancreatic, hepatic and gastric cancer in our view clearly indicate that this B7-H3xCD3 bsAb constitutes a promising immunotherapeutic compound. GMP compliant production of CC-3 is presently ongoing to enable clinical evaluation.

## Data availability statement

The raw data supporting the conclusions of this article will be made available by the authors, without undue reservation.

## Ethics statement

The studies involving human participants were reviewed and approved by IRB (ethics committee of the Faculty of Medicine of the Eberhard Karls Universitaet Tuebingen) at the University Hospital Tuebingen and was conducted in accordance with the Declaration of Helsinki; reference number13/2007 V. Human material was collected after obtaining informed consent. The patients/participants provided their written informed consent to participate in this study.

## Author contributions

ML designed and performed the experiments, analyzed and interpreted data, and wrote the manuscript. LZ designed experiments, provided critical reagents and contributed to the study design. LW designed and performed experiments. GJ, SB and UL provided critical reagents. JH wrote and critically revised the manuscript. HS contributed to the study design, critically revised the manuscript, and supervised the study. All authors contributed to the article and approved the submitted version.
