# Supplementary material for: IgG-based B7-H3xCD3 bispecific antibody for treatment of pancreatic, hepatic and gastric cancer
Source: Front Immunol. 2023 Apr 14;14:1163136. doi: 10.3389/fimmu.2023.1163136 (PMC10140336; doi:10.3389/fimmu.2023.1163136)
Supplement: Supplementary file 1 [file DataSheet_1.docx]

Supplementary Figures


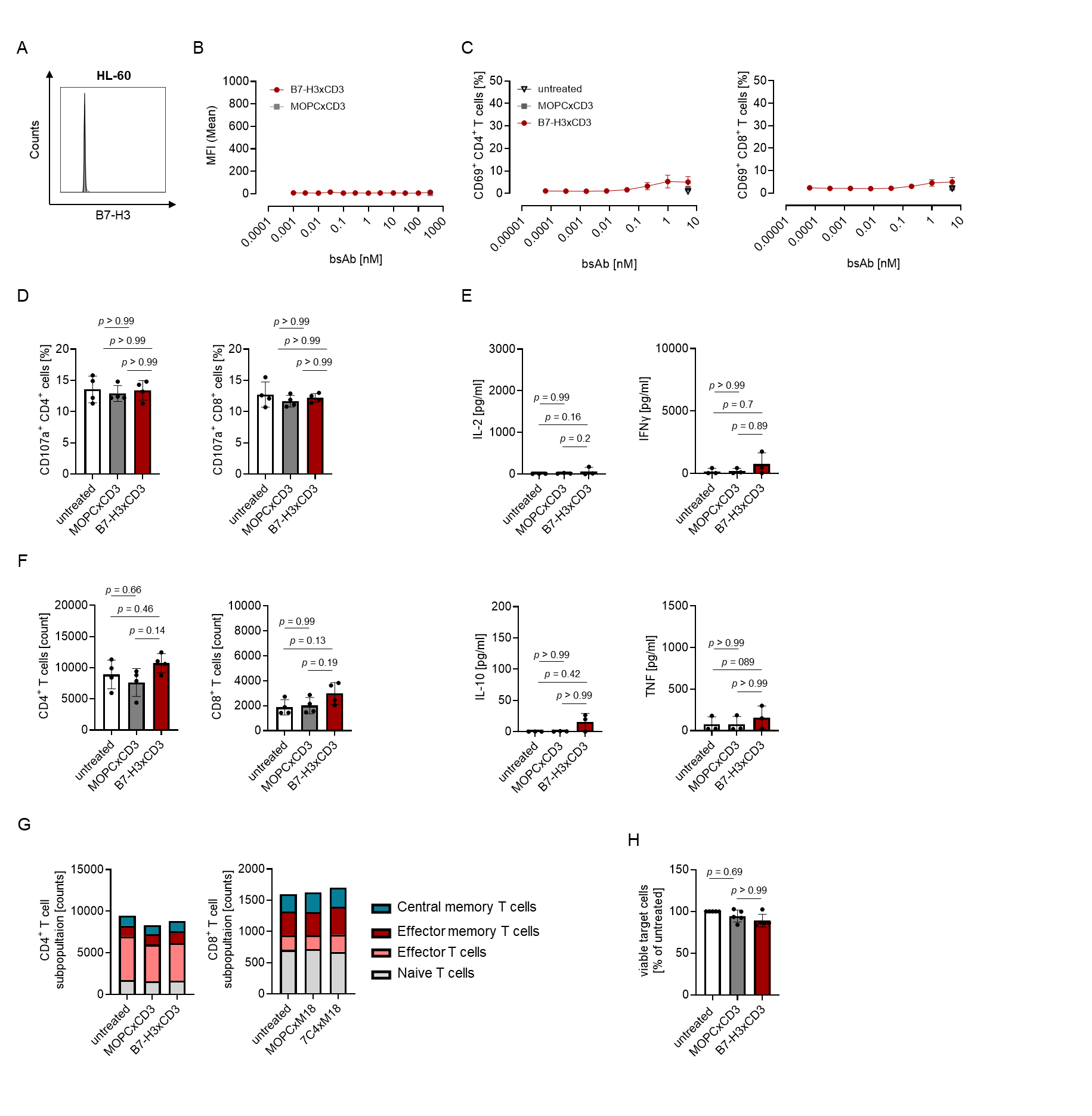


**Figure S1: Dependence of CC-3 activity on target antigen-binding. A** HL-60 cells were stained using a monoclonal B7-H3 antibody (clone 7C4) followed by an anti-mouse PE conjugate and analyzed using flow cytometry, documenting negativity for B7-H3. **B** HL-60 cells were incubated with increasing concentrations of the B7-H3xCD3 bsAb or the respective isotype control MOPCxCD3, followed by an anti-human PE conjugate and analyzed by flow cytometry, documenting absence of unspecific binding. **C-H** Monocyte-depleted PBMC of healthy donors were incubated with the B7-H3 negative HL-60 cells in the presence or absence of CC-3 or MOPCxCD3. If not indicated otherwise, constructs were used at 1 nM. **C** Activation of CD4^+^ and CD8^+^ T cells (E:T 4:1) was determined by flow cytometric analysis for CD69 expression after 24 hours. Combined data obtained with PBMC of three independent donors are shown. **D** Degranulation of CD4^+^ and CD8^+^ T cells (E:T 4:1) was determined by analyzing expression of CD107a after 4 h. Combined data obtained with PBMC of four independent donors are shown. **E** IL-2, IFNγ, IL-10 and TNF levels in culture supernatants were measured after 24 h using LEGENDplex assays. **F-G** After 72 h, PBMC were reexposed to fresh HL-60 cells and the respective treatment for additional 72 h. **F** Proliferation of CTV labelled CD4^+^ and CD8^+^ T cells (E:T 4:1) was determined by flow cytometric analysis of cell counts. **G** Subpopulations within CD4^+^ and CD8^+^ T cells were determined by flow cytometric analysis. Effector T cells were defined as CD62L^-^CD45ro^-^, naive T cells as CD62L^+^CD45ro^-^, effector memory T cells as CD62L^-^CD45ro^+^ and central memory T cells as CD62L^+^CD45ro^+.^ **H** Lysis of HL-60 cells was determined by flow cytometric analysis. E:T, effector to target; MFI, mean fluorescence intensities; PBMC, peripheral blood mononuclear cell.


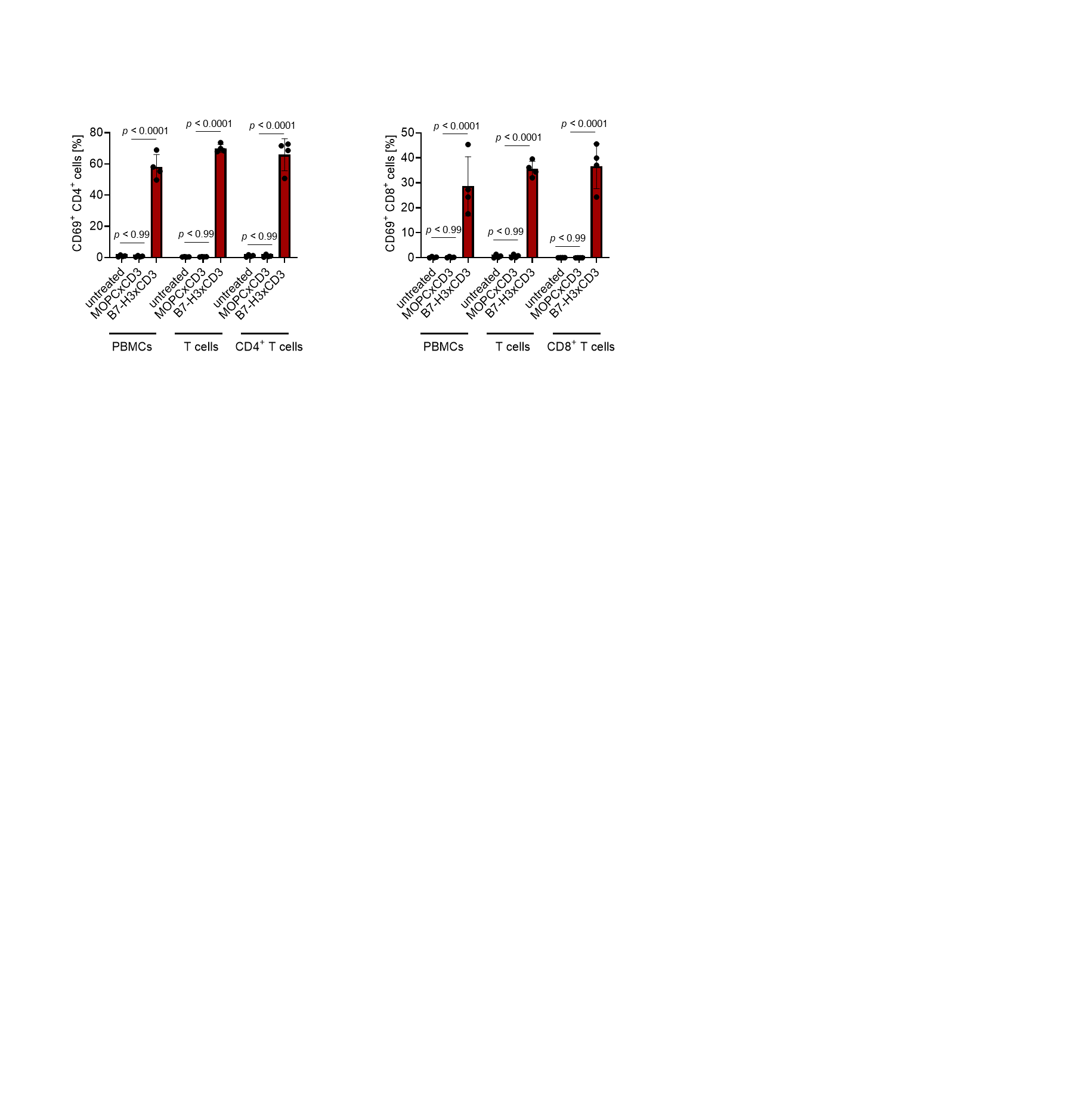


**Figure S2: Activation of T cells within PBMCs and isolated T cell subsets by CC-3.** Recombinant B7-H3 protein was immobilized to plastic and incubated with PBMC preparations or isolated T cells of healthy donors and MOPCxCD3 or CC-3 (5nM each). Activation of CD4^+^ and CD8^+^ T cells was determined by flow cytometric analysis for CD69 expression after 72 h. PBMC, peripheral blood mononuclear cells.


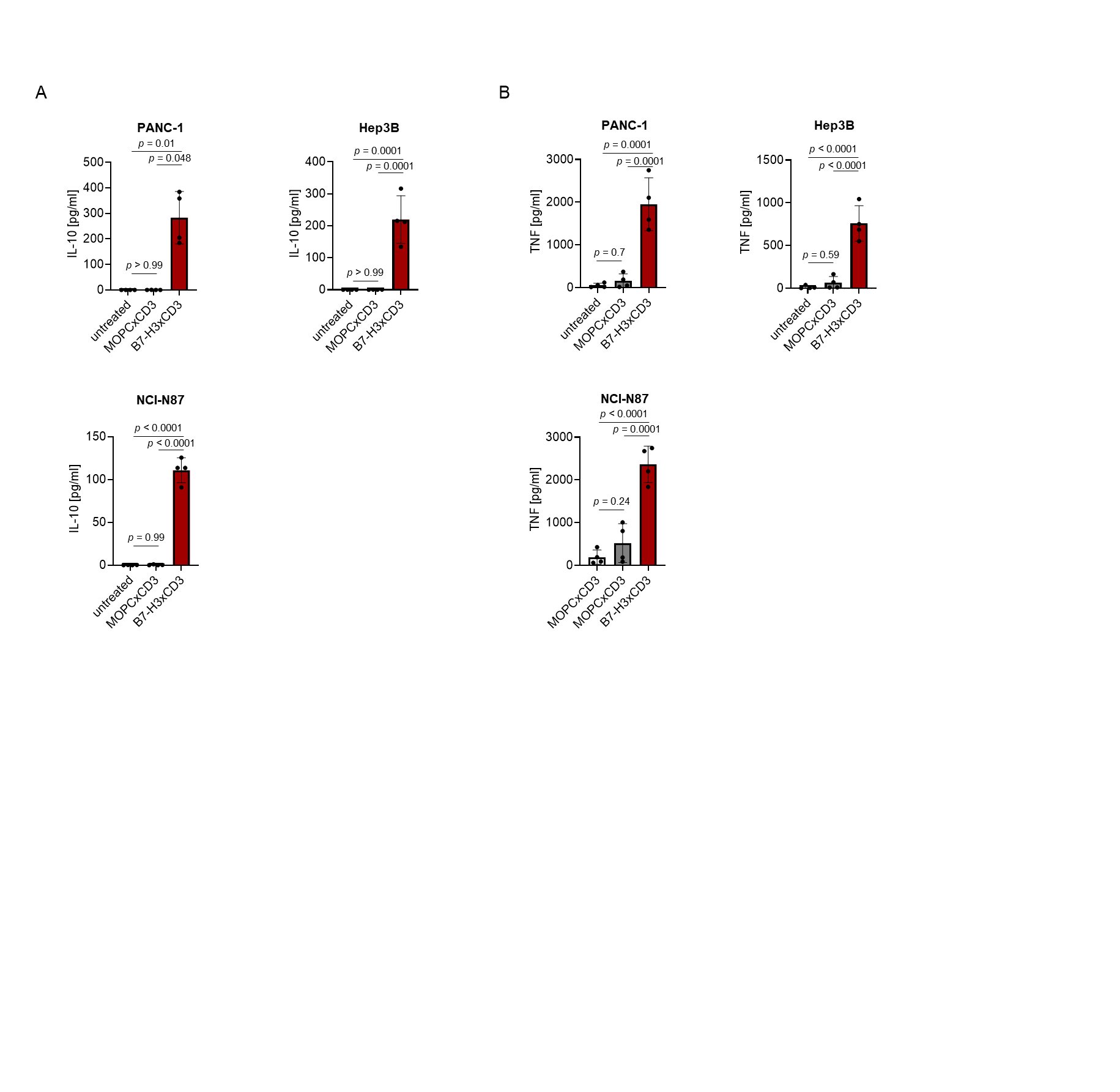


**Figure S3: Induction of cytokine secretion by CC-3.** Monocyte-depleted PBMC of healthy donors were incubated with the indicated tumor cell lines (E:T 4:1) in the presence or absence of CC-3 or MOPCxCD3 (1 nM each). IL-10 and TNF levels in culture supernatants were measured after 24 h using LEGENDplex assays. Combined data obtained with PBMC of four independent donors are shown. E:T, effector to target; PBMC, peripheral blood mononuclear cell.


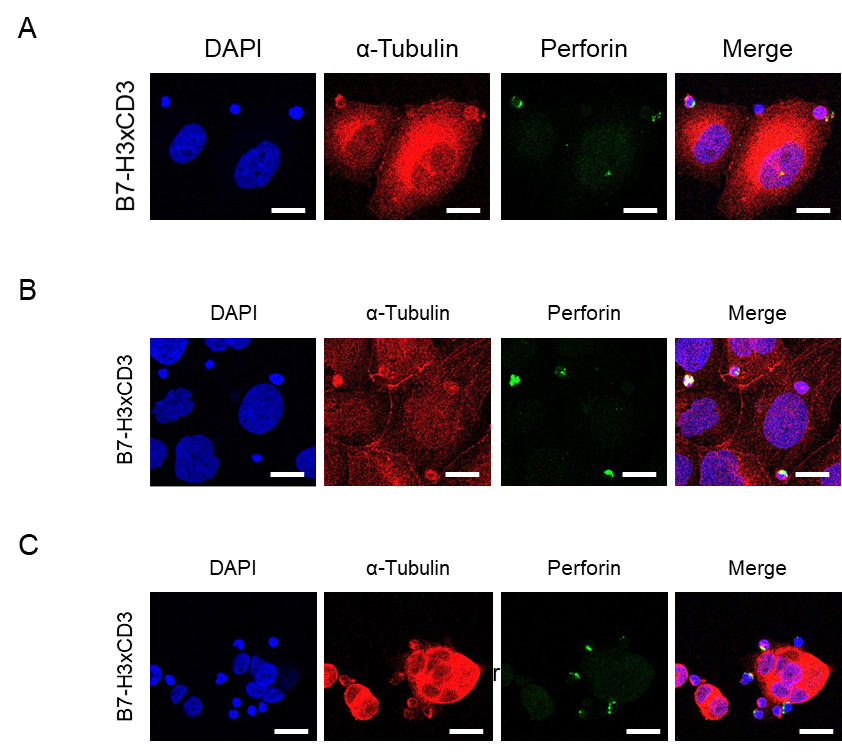


**Figure S4 Induction of Perforin expression by CC-3**. Monocyte-depleted PBMC of healthy donors were incubated with the target cell lines **A** PANC-1, **B** Hep3B or **C** NCI-N87 (E:T 4:1) in the presence or absence of CC-3 or MOPCxCD3 (1 nM each) for 3 h and subsequently stained for Perforin (green) and α-Tubulin (red). DAPI was used for counterstaining. Scale bars indicate 20 µm, original magnification x63. DAPI, 4′,6-diamidino-2-phenylindole; E:T, effector to target.


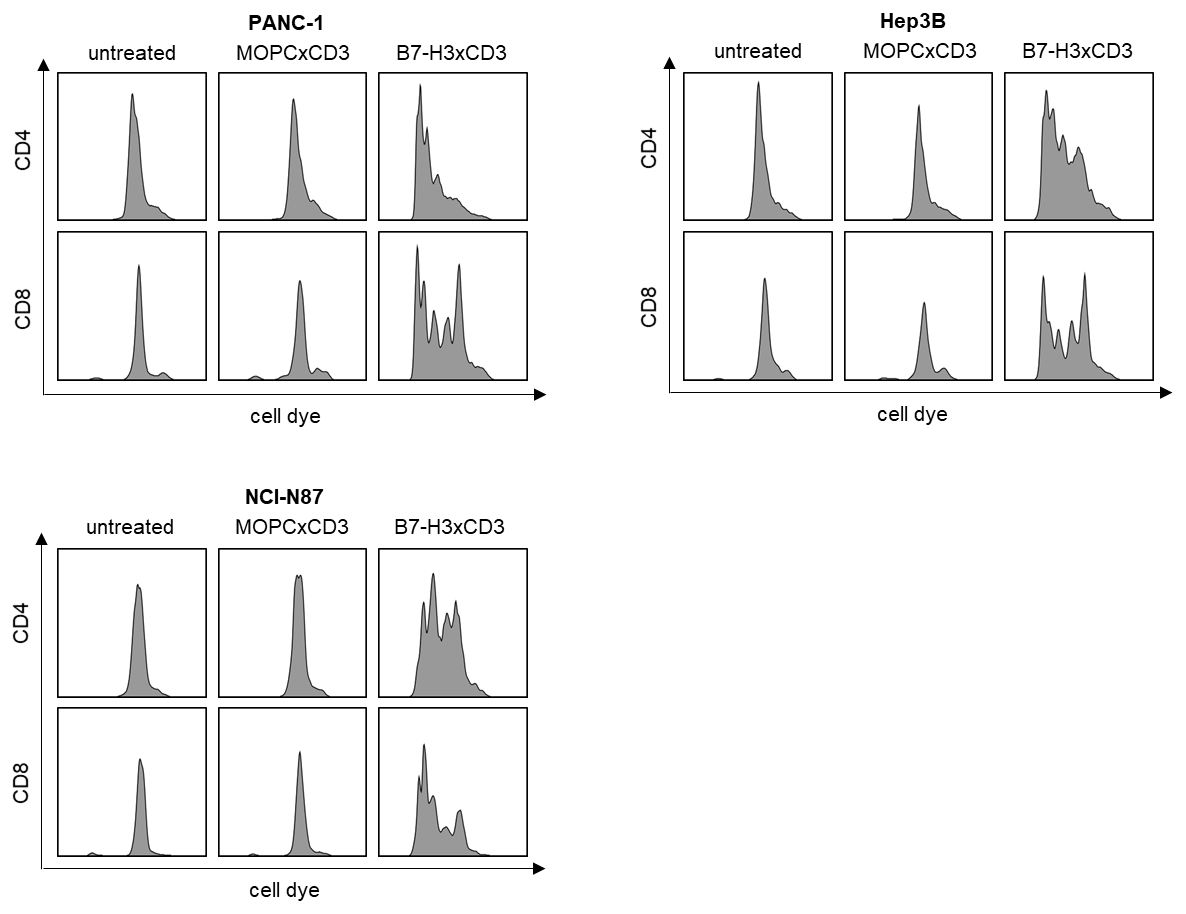


**Figure S5 Induction of T cell proliferation by CC-3.** Monocyte-depleted PBMC of healthy donors (n=4) were labelled with CellTrace^TM^ Violet cell dye and incubated with or without MOPCxCD3 or CC-3 (1 nM each) in the presence of the indicated target cell lines (E:T 4:1). After 72 h, cells were reexposed to fresh target cells and the respective treatment. After additional 24 h, cell dye dilution was determined by flow cytometry. E:T, effector to target; PBMC, peripheral blood mononuclear cell.


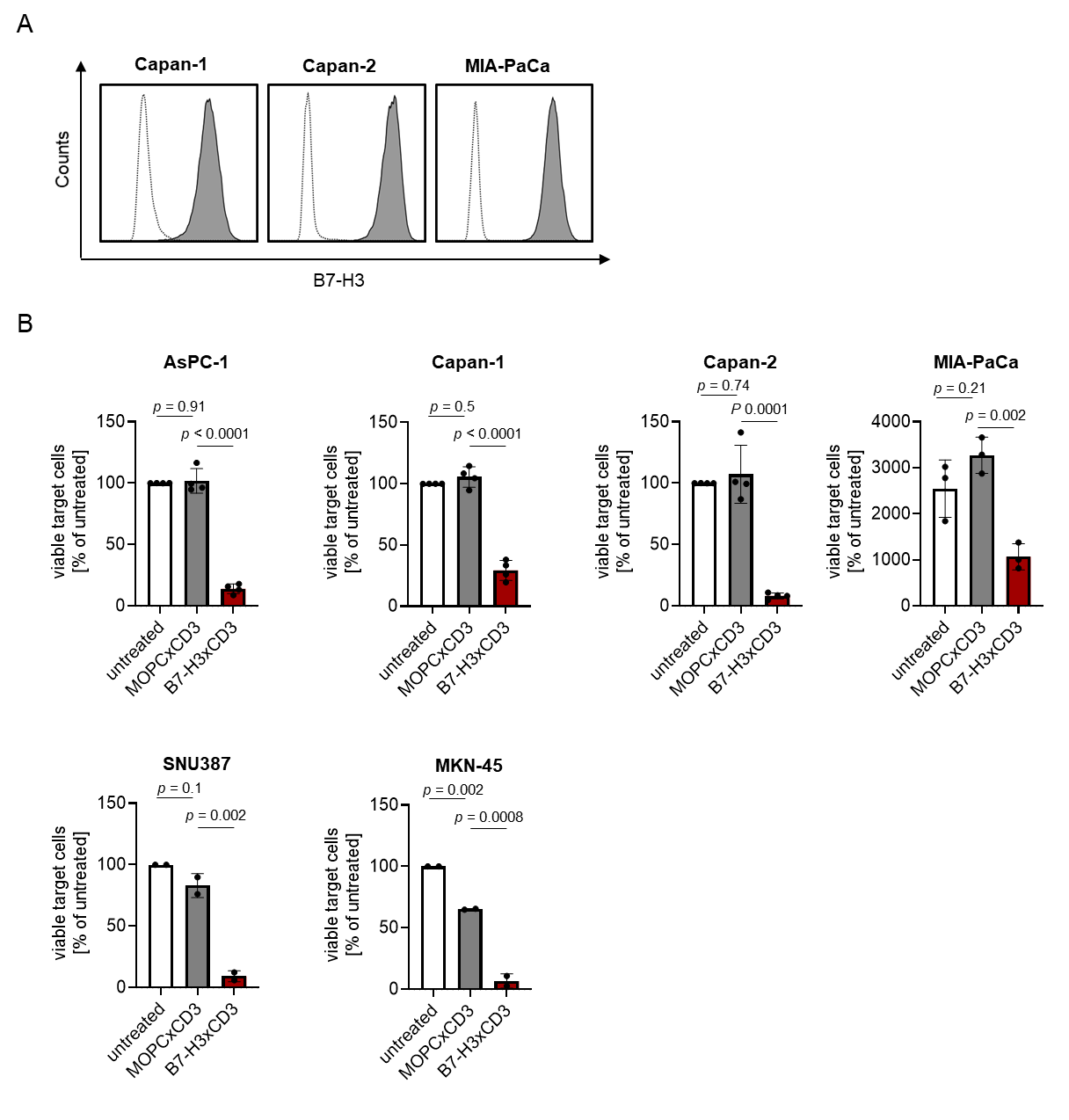


**Figure S6 Extended functional characterization of CC-3. A** The indicated cancer cell lines were stained using a murine monoclonal B7-H3 antibody (clone 7C4) followed by an anti-mouse PE conjugate and analyzed using flow cytometry. B7-H3 expression on pancreatic cell lines Capan-1, Capan-2 and MIA-PaCa is shown (shaded peaks, anti-B7H3; open peaks, control). **B**Monocyte-depleted PBMC of healthy donors (at least 2 donors) were incubated with the indicated target cells at an E:T ratio of 4:1 in the presence or absence of MOPCxCD3 or CC-3 (1 nM each). Lysis of Capan-1, Capan-2, MIA-PaCa, SNU387 and MKN-45 was determined by flow cytometry-based lysis assay after 72 h. E:T, effector to target; PBMC, peripheral blood mononuclear cells.
